# Altered Cerebellar Biochemical Profiles in Infants Born Prematurely

**DOI:** 10.1038/s41598-017-08195-4

**Published:** 2017-08-15

**Authors:** Marie Brossard-Racine, Jonathan Murnick, Marine Bouyssi-Kobar, Janie Coulombe, Taeun Chang, Catherine Limperopoulos

**Affiliations:** 1 McGill University Health Centre, Division of Pediatric Neurology, Montreal, PQ Canada; 2 School of Physical and Occupational Therapy, McGill University, Montreal, PQ Canada; 3 Division of Diagnostic Imaging and Radiology, Children’s National Health System, Washington, D.C., USA; 4 Developing Brain Research Laboratory, Children’s National Health System, Washington, D.C., USA; 5 Epidemiology, Biostatistics and Occupational Health, McGill University, Montreal, PQ Canada; 6 Neurophysiology, Epilepsy and Critical Care, Children’s National Health System, Washington, D.C., USA

## Abstract

This study aims to compare the cerebellar biochemical profiles in preterm (PT) infants evaluated at term equivalent age (TEA) and healthy full-term newborns using proton magnetic resonance spectroscopy (^1^H-MRS). We explore the associations between altered cerebellar metabolite profiles and brain injury topography, severity of injury, and prematurity-related clinical complications. We prospectively collected high quality ^1^H-MRS in 59 premature infants born ≤32 weeks and 61 healthy full term controls. ^1^H-MRS data were processed using LCModel software to calculate absolute metabolite concentration for N-acetyl-aspartate (NAA), choline (Cho) and creatine (Cr). PT infants had significantly lower cerebellar NAA (p < 0.025) and higher Cho (p < 0.001) at TEA when compared to healthy controls. Creatine was not different between the two groups. The presence of cerebellar injury was consistently associated with reduced concentrations for NAA, Cho, and Cr. Postnatal infection was negatively associated with NAA and Cr (p < 005), while cerebral cortical brain injury severity was inversely associated with both Cho and Cr (p < 0.01). We report for the first time that premature birth is associated with altered cerebellar metabolite profiles when compared to term born controls. Infection, cerebellar injury and supratentorial injury are important risk factors for impaired preterm cerebellar biochemistry.

## Introduction

Over the past fifteen years, neuroimaging studies have brought to the forefront a previously underrecognized prevalence of cerebellar growth impairment following preterm (PT) birth^1^. Conventional imaging studies first described a high incidence of structural cerebellar injury (CBI) in survivors of PT birth^2–4^. With the introduction of quantitative magnetic resonance imaging (MRI), secondary arrested growth of the cerebellum in the setting of severe, isolated supratentorial injury (SPI), has been demonstrated in the cerebellum, suggesting trophic transsynaptic effects of the cerebro-cerebellar pathway^5–7^. Even in the absence of supra- or infratentorial lesions on conventional MRI, cerebellar underdevelopment or hypoplasia has also been reported in survivors of prematurity during adolescence and adulthood^8, 9^. This underdevelopment may be suggestive of genetic or chromosomal anomalies^10^ but more than likely reflects microscopic injury that is below the current level of resolution of conventional MRI.

Proton magnetic resonance spectroscopy (^1^H-MRS) is a non-invasive imaging technique that measures *in vivo* concentrations of brain tissue metabolites. Common brain metabolites that can be accurately detected include; N-acetyl-aspartate (NAA), choline (Cho) and creatine (Cr). NAA is a marker of neuronal integrity^11^, and a reduction in concentration is associated with neuronal injury and immaturity^12^. Cho is a marker of membrane structural integrity and membrane turn over, and Cr is a marker of energy stores. Decreased concentration of both of these metabolites would indicate altered neuronal integrity^13^. Together, these metabolites provide important biochemical information on the integrity of the developing neuronal tissue. Cerebral metabolites evaluated in preterm infants at term equivalent age (TEA) have shown to be a promising biomarker of later neurodevelopment outcomes^14^. However, cerebellar biochemical profiles remain to be examined. The objective of this study was to compare the cerebellar biochemical profiles in PT infants evaluated at TEA to healthy full-term newborns using ^1^H-MRS, and to investigate associations between cerebellar metabolite profiles, brain injury topography (i.e. infra- and supratentorial) and severity, and prematurity-related clinical complications.

## Results

A total of 210 cerebellar ^1^H-MRS acquisitions were acquired in 103 full-term controls and 94 PT infants at TEA. Of these, only 125 spectra (59%) met our rigorous quality assessment criteria and were included in our final analyses. These included, 61 spectra from 61 controls (mean gestational age (GA) = 41.55 weeks) and 64 spectra from 59 PT infants (mean GA = 39.91weeks) of whom five PT infants had two MRIs at TEA. All controls had a normal structural brain MRI. Of the 59 PT infants, 24 had no parenchymal injury, 23 had SPI only, 10 had both SPI and CBI, and 2 had CBI only. Intraventricular hemorrhage (IVH) was seen in 20 PT infants: 5 grade I, 7 grade II, 1 grade III, 7 grade IV. Detailed clinical and demographic characteristics are presented in Table 1. Excluded subjects did not differ from the rest of the sample with respect to birthweight, gestational age at birth or at time of MRI, Apgar score, and MR injury severity score.

**Table 1:** Clinical characteristics of the cohort (N = 120).

	Term (n = 61)	Preterm (n = 59)
	Mean ± SD/Median[range]	Mean ± SD/Median[range]
**Ante and neonatal**		
Clinical chorioamnionitis n (%)	0 (0%)	14 (23.7)
GA at birth (weeks)**	39.6 ± 1.2	26.7 ± 2.7
Birth weight (grams)**	3321 ± 364	872 ± 334
Female n (%)	28 (45.9)	33 (55.9)
Intubation at birth n (%)	0 (0%)	27 (48.2)
Apgar score 1 min**/5 min**	8[6–9]/9[8–9]	4 [1–9]/7[1–9]
Patent ductus arteriosus ligation n (%)	NA	19 (32.2)
Surgery for necrotizing enterocolitis n (%)	NA	29 (49.2)
Neonatal infection n (%)	NA	21 (35.6)
Days on mechanical ventilation	NA	30 ± 38
Days on supplementary oxygen	NA	86 ± 51
Chronic lung disease n (%)	NA	38 (64.4%)
Days in NICU	NA	120 [30–205]
**Neonatal MRI**		
Gestational age at MRI (weeks)**	41.5 [38.3–45.6]	39.9 [35.3–44.4]
Day of life at MRI**	12 [4–31]	96 [30–138]
White matter injury score	NA	3 [0–16]
Combined grey matter injury score	NA	1 [0–9]
Cerebellar score	NA	1 [0–7]

Legend: **p < 0.01.

Mean differences in metabolite absolute concentrations between the two groups are summarized in Table 2. When controlling solely for GA at MRI, cerebellar total NAA (tNAA) was significantly lower (p = 0.025) and total Cho (tCho) was significantly higher (p < 0.001) in PT infants when compared to the controls. However, total Cr (tCr) was not significantly different between groups. After accounting for cerebellar signal abnormality (i.e. CBI) in the model, although metabolic differences showed the same trend, only tCho alterations remained significant (p < 0.001).

**Table 2:** Absolute concentrations of brain metabolite in preterm and healthy full-term controls (Mean ± SD)^1^.

Metabolites^2^	Preterm	Control	P-values
tNAA	6.817 ± 1.936	8.282 ± 1.755	0.025
tCho	6.012 ± 1.295	5.434 ± 0.737	<0.001
tCr	9.608 ± 2.110	10.200 ± 1.721	0.693

Legend: ^1^controlling for GA at MRI; ^2^concentration is in mM/kg.

The best forward multiple linear regression models evaluating associations between clinical complications and metabolites in the PT infants are presented in Table 3. GA at MRI and the presence of CBI were respectively associated with increased and reduced concentration in all three metabolites. Infection was negatively associated with tNAA and tCr, while the combined grey matter injury severity was inversely associated with both tCho and tCr. No other significant associations were found among any other clinical factors.

**Table 3:** Best forward multiple linear regression models of clinical complications associated with cerebellar metabolites^1^.

Metabolites	R2 (models’ p-values)	Variables	Standardized coefficients	Variables’ p-values
tNAA	0.315 (<0.001)	GA	0.474	<0.001
		Infection	−0.247	0.042
		CBI	−0.298	0.012
tCho	0.356 (<0.001)	GA	0.384	0.001
		CBI	−0.349	0.002
		Combined grey matter injury	−0.381	0.001
tCr	0.514 (<0.001)	GA	0.641	<0.001
		CBI	−0.706	<0.001
		Infection	−1.075	0.015
		Combined grey matter injury	−0.273	0.017

Legend: ^1^in mM/kg; GA, gestational age at MRI; CBI, cerebellar injury.

## Discussion

Our study reports altered cerebellar metabolite profiles in prematurely born infants at TEA when compared to healthy term controls. Alterations in absolute cerebellar metabolite concentrations were primarily observed in NAA and Cho but not in Cr. As expected, absolute concentrations of all three metabolites were highly associated with CBI. The only other clinical risk factors associated with biochemical impairments in the cerebellum were severity of supratentorial grey matter injury and neonatal infection.

To date, there is very limited published data to compare our cerebellar metabolic results. Only one previous study attempted to compare absolute cerebellar metabolite concentrations in a small subset of PT infants and full term controls with limited success due to the small number of spectra successfully obtained in this region (4 PT vs. 7 controls)^15^. Our study is the first to provide evidence for altered cerebellar metabolism in a group of PT infants compared to an equally large number of healthy full-term controls.

In our study, we successfully obtained spectra in 12 PT infants with direct cerebellar injury. These injuries were overall mild, given the inherent difficulty of acquiring good quality spectra in the presence of extensive injury. The inclusion of too much cerebrospinal fluid (CSF) and/or residual blood product in the voxel of interest usually dominates the tissue’s signal and results in a failed acquisition. Similarly, when residual cerebellar volume is too little, the small voxel size used to capture the ^1^H-MRS does not provide sufficient signal-to-noise ratio needed to successfully obtain good quality spectra. In this subset of 12 PT infants, we found that cerebellar injury was associated with decreased NAA, Ch, and Cr. Reduced NAA is known to be a marker of altered neurogenesis or neuronal loss^16, 17^. Our data suggests that in the presence of CBI, cerebellar neuronal damage extends beyond the site of the injury and is present in regions apparently intact as evaluated by conventional MRI. Available evidence in PT infants suggests that lower cerebellar NAA/Cho ratio evaluated at TEA is associated with lower cognitive scores at 2 years of age^18^. However, an understanding of the true contribution of NAA is limited given that it cannot be separated from the variation of Cho, the denominator in ratio analyses^19^. We show for the first time in our study that absolute concentrations of Cho are not constant in PT infants, as Cho is affected by both CBI and SPI. Our findings therefore emphasize the limitation of using ratio analyses for interpreting metabolic disturbances particularly in the PT infant.

Our findings demonstrate that only Cho remained significantly higher in the PT group after controlling for GA at MRI and CBI. During typical cerebral development, choline concentration reaches its highest values in the first few months of life and steadily decreases after the relative completion of the myelin’s most important formation phase that is characteristically before 2 years of age^13, 20^. We previously reported reduced mean diffusivity in the vermis of PT without CBI when compared to healthy controls reflecting a possible increase in cellular density^21^. Together with our observation of increased Cho in PT by TEA, these data suggest a possible cerebellar cellular/microstructural overgrowth or overexpression which may represent a compensatory mechanism triggered by an interruption of the cerebellum preprogrammed developmental course. Notably, cerebral and cerebellar overgrowth, thought to be a consequence of apoptosis failure have been previously reported in developmental disorders including fetal ventriculomegaly^22^, postnatal hydrocephalus^23^ and autism^24, 25^. However, in the absence of cerebellar volumetric analyses and serial prospective follow-up studies, interpretation of our cross-sectional observations remains speculative.

In addition to CBI, which consistently mediated all three metabolites in the PT cohort, combined supratentorial grey matter injury was also associated with lower Cho and Cr concentrations. Interestingly, the white matter injury score was not associated with lower Cho and Cr concentrations. This association may in part be explained by the fact that the PT infants in our sample had less overall cortical or subcortical grey matter abnormalities as compared to the frequent mild white matter abnormalities previously described^26^. Negative associations between Cho, Cr and cerebral cortical grey matter injury further describe the cerebro-cerebellar diaschisis effects on cerebellar development. Secondary growth impairment of the cerebellum in the presence of severe SPI has been previously described^5–7^. This study adds to the existing literature by demonstrating disturbances in cerebellar metabolism in the preterm infant.

Our data showed that postnatal infection is also negatively associated with NAA and Cr in PT infants. Through elevated cytokine responses, maternal and neonatal infections are thought to be an important etiologic factor for periventricular white matter injury^27^. The negative effect of infection on cerebral NAA/Cho ratio has been previously described in the cerebrum of PT infants at TEA even in the absence of parenchymal injury^28^. However, this neurotoxic response had not been previously described in the cerebellum. Our relatively modest sample size may have limited our ability to detect more complex associations and their interactions with other risk factors described in our cohort.

Our study limitations deserve mention. Our 60% success rate in performing spectroscopy was somewhat low, but comparable between our control and PT infant cohorts. The overall baseline characteristics (e.g. birthweight, age at MRI) for excluded or failed ^1^H-MRS subjects did not differ from the rest of the sample. We therefore believe that our reduced success rate is explained in part by the fact that our ^1^H-MRS acquisition was at the end of our MRI protocol. This acquisition is particularly sensitive to motion. As our MRIs were performed during natural sleep (without sedation), we had several babies who woke up before or during the MRI, preventing us from completing the ^1^H-MRS acquisition. When interpreting our results, it is important to note that we have calculated our absolute concentration values assuming that our voxel only contained cerebellar white matter. Although we made sure that our voxel predominantly contained cerebellar white matter, due to the extensive foliation of the cerebellum, the position of its deep nuclei and its overall shape, we cannot overlook the possibility that our voxels may have been in part contaminated by a small quantity of grey matter or CSF.

In summary, this study provides the first evidence for altered cerebellar metabolic profiles in PT infants at TEA when compared to healthy term born infants. In keeping with the already well-documented cerebellar disturbances observed in the PT infants, our findings provide evidence for *in vivo* metabolic disturbances at a biochemical level. Ongoing studies with larger sample sizes and longitudinal follow-ups are needed to better understand how these metabolic disturbances relate to the developmental cerebellar cognitive affective syndrome frequently observed in surviving preterm infants.

## Methods

### Participants

This is a prospective study of premature infants born ≤32 weeks and of ≤1500 g birth weight, admitted to a tertiary neonatal intensive care unit (NICU) at Children’s National Health System (CNHS) (Washington, D.C.) between June 2012 and February 2016. PT infants with congenital malformations or dysmorphic features suggestive of a genetic syndrome, chromosomal abnormality, confirmed inborn errors of metabolism or central nervous system infection were excluded. Full-term control infants were recruited from a parallel study evaluating serial brain development in utero and postnatally in healthy full term infants (≥37 weeks) born without event to healthy singleton pregnant volunteers^29^. Volunteers with abnormal findings on either fetal ultrasound or postnatal brain MRI of their newborns were excluded. Ante-, peri- and postnatal information for all enrolled subjects were collected through review of medical records and parental questionnaires. Written informed consent was obtained for every participant, and the study was approved by the CNHS Institutional Review Board. All aspects of the study were performed in accordance with relevant institutional guidelines and regulations.

### MRI acquisitions

Unless clinically indicated for PT infants, no sedation or intravenous injection of contrast agents were used during the infant’s MRIs. The non-sedated MRIs were performed during natural sleep after feeding and swaddling the infants. All MRIs were performed on a 3 Tesla MRI scanner (Discovery MR750, General Electric Medical Systems-Waukesha, WI) with an 8-channel receiver head coil. PT infants were scanned when medically stable at TEA with a targeted GA window of 39–41 weeks. Healthy control newborns completed their brain MRIs as outpatients without sedation, shortly after birth.

As part of our multimodal MRI acquisition protocol, structural images (T2 3D-cube and T1 3D-SPGR) and ^1^H-MRS were acquired using the exact same protocol for all preterm and full-term control infants. Each brain MRI study was reviewed by an experienced pediatric neuroradiologist (J.M). MRI abnormalities were scored using the Kidokoro *et al*.^30^ scoring system for PT infants at TEA. This scoring system uses 13 items to evaluate brain maturity and injury of the cerebral white matter, cerebral cortical and deep grey matter as well as the cerebellum which together form the total injury score. Direct CBI was characterized by the presence of signal abnormality, which corresponds to a score >0 on the cerebellar signal item. In an attempt to capture the impact of injury localization, we used the cerebellar, white, and grey matter sub-scores separately in our analyses rather than the total score. Due to the relative small number of injured PT infants, we combined cortical and deep grey matter (Kidokoro’s subscale) to obtain an overall grey matter sub-score. Germinal matrix and intraventricular hemorrhages were separately graded according to Papile’s grading system^31^.

**Figure 1:**
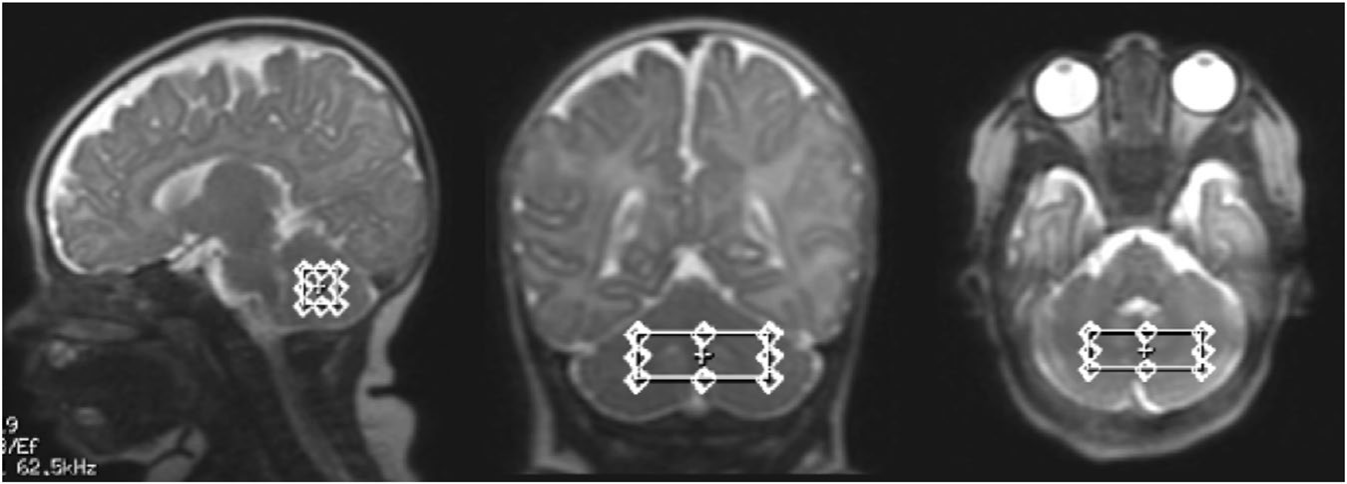
Example of a single ^1^H-MRS voxel placed in the middle of the cerebellum on corresponding sagittal, coronal and axial T2 images.

**Figure 2:**
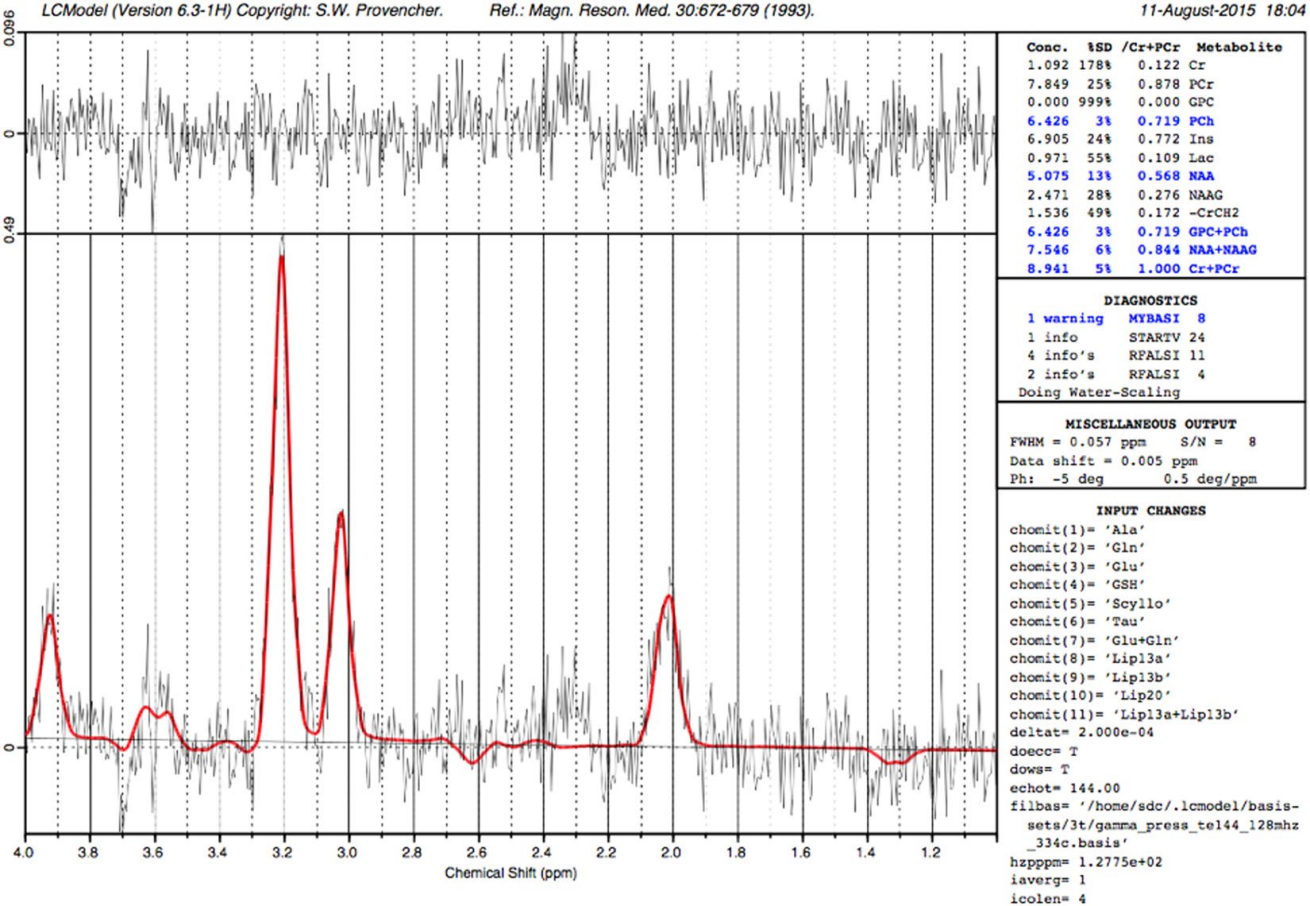
Typical good quality LCmodel spectra output obtained in a preterm infant at 38.14 weeks of age.

**Figure 3:**
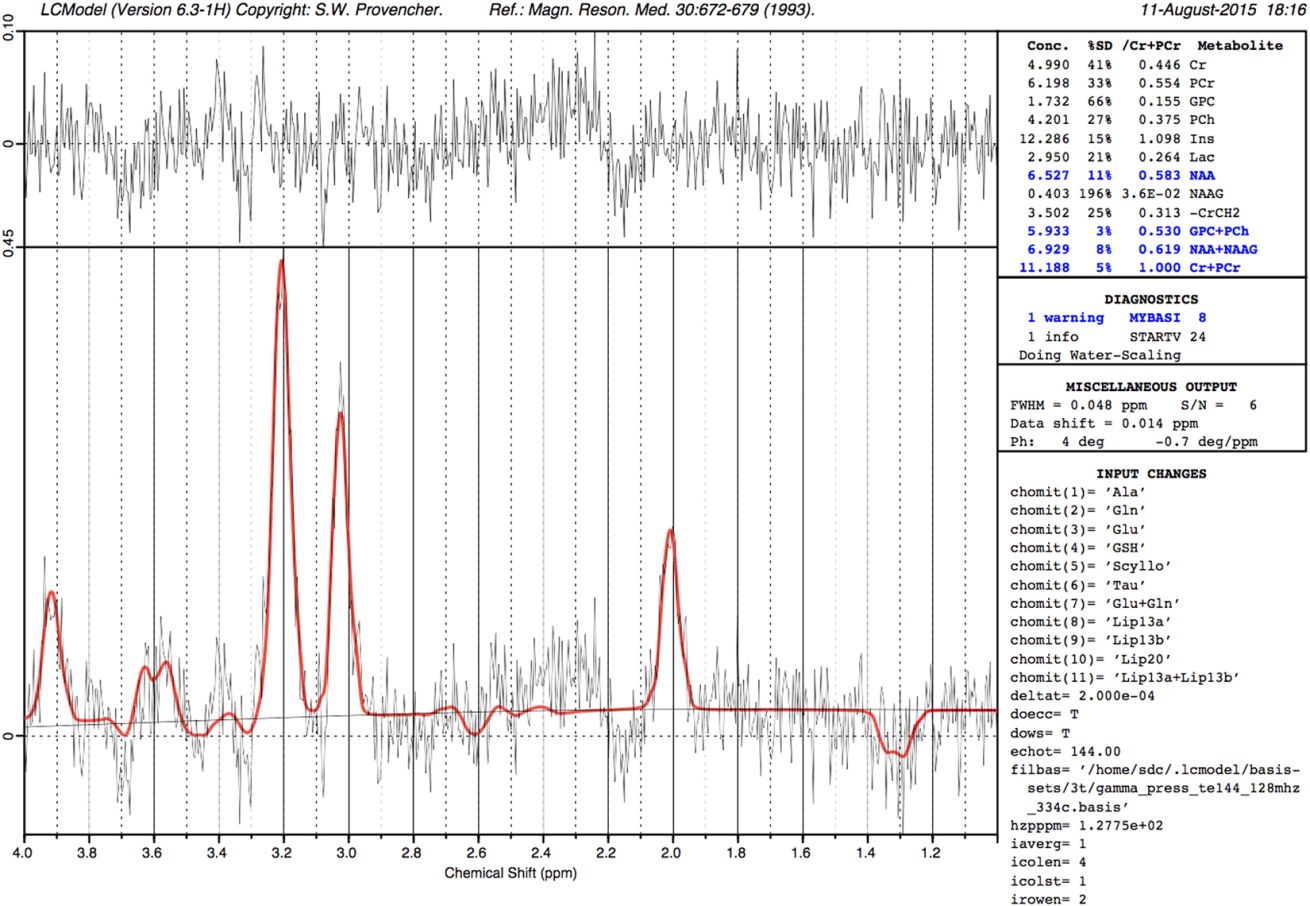
Typical good quality LCmodel spectra output obtained in a healthy control at 39.43 weeks of age.

^1^H-MRS were acquired from a single voxel of 3 cm3 (1 × 3 × 1) on average placed in the middle of the cerebellum avoiding the skull and extra-axial spaces (Fig. 1), using a point resolved spectroscopy (PRESS) sequence with a TE of 144 ms, a TR of 1500 ms, and 128 signal averages. Two additional oblique outer volume suppression pulses were placed to minimize unwanted signals from outside the voxel and flow artifacts from the vasculature and CSF^1^.H-MRS data were processed using LCModel software^32^ to calculate absolute metabolite concentrations in the chemical shift range of 4.0 to 1.0 ppm, using the unsuppressed water signal as an internal reference. Figures 2 and 3 present examples of our processed spectra output. We assumed that the water absolute concentration was constant in the evaluated tissue and that the voxel contained only white matter. Absolute metabolite concentrations were then corrected for tissue water content estimated in the cerebellum around term (88%) and calculated in mM/kg^33^.

Individual metabolite spectra were then generated for the six following compounds: Creatine (Cr), Phosphorecreatine (PCr), Glycerophosphorylcholine (GPC), Phosphocoline (PCh), N-Acetyl-acetylaspartate (NAA), and N-acetyl-aspartyl-glutamate (NAAG). The sums of the absolute metabolite concentrations [tNAA = NAA + NAAG, tCho = GPC + PCh, tCr = PCr + Cr] were used in the analysis since they represent more accurate estimates. Based on the LCModel output, MR spectra with a line width at FWHM greater than 0.129 ppm and/or signal to noise ratio of less than three were excluded.

### Statistical analysis

Descriptive statistics for both groups were performed first. Thereafter, replicates for individuals who had more than one ^1^H-MRS dataset were randomly excluded and only one observation was kept such that the whole set was made of independent observations. All subsequent analyses were performed on that subset. To assess the difference in cerebellar metabolite concentrations between the cases and controls, analysis of covariance was performed adjusting for GA at time of MRI and then for the presence of cerebellar signal abnormality. Subsequently, we wanted to explore the effect of brain injury topography and other clinical complications on cerebellar metabolites. In the PT group only, partial regressions were first performed in order to assess which variables were significantly associated to each metabolite after adjusting for GA at MRI. Univariate analyses explored associations between each metabolite and the following variables: histologic chorioamnionitis, GA at birth, intubation at birth, Apgar score at 5 minutes of life, surgery for necrotizing enterocolitis or for patent ductus arteriosus, clinical infection as determined by positive cultures or by treatment with antibiotics for more than 7 days, highest respiratory support, length of stay on oxygen, length of NICU admission, and the three different subscores for brain injury (i.e. combined grey matter injury, subcortical white matter injury, cerebellar injury). For each metabolite, a forward regression was then performed, in which all significant risk factors found at the previous step were used as covariates along with GA at MRI. The final models represented the best sets of risk factors, which were the most linked to each metabolite outcome. We used a 5% level of significance. All the analyses were done with SPSS v19.

## References

[1] Brossard-Racine M, du Plessis AJ, Limperopoulos C (2015). Developmental cerebellar cognitive affective syndrome in ex-preterm survivors following cerebellar injury. Cerebellum.

[2] Limperopoulos C (2005). Cerebellar hemorrhage in the preterm infant: ultrasonographic findings and risk factors. Pediatrics.

[3] Limperopoulos C, Robertson RL, Sullivan NR, Bassan H, du Plessis AJ (2009). Cerebellar injury in term infants: clinical characteristics, magnetic resonance imaging findings, and outcome. Pediatr Neurol.

[4] Steggerda SJ (2009). Cerebellar Injury in Preterm Infants: Incidence and Findings on US and MR Images. Radiology.

[5] Srinivasan L (2006). Smaller Cerebellar Volumes in Very Preterm Infants at Term-Equivalent Age are Associated with the Presence of Supratentorial Lesions. American Journal of Neuroradiology.

[6] Limperopoulos C (2005). Impaired Trophic Interactions Between the Cerebellum and the Cerebrum Among Preterm Infants. Pediatrics.

[7] Tam EW (2009). Cerebellar development in the preterm neonate: Effect of supratentorial brain injury. Pediatr Res.

[8] Parker J (2008). Cerebellar growth and behavioural & neuropsychological outcome in preterm adolescents. Brain.

[9] Allin M (2001). Cognitive and motor function and the size of the cerebellum in adolescents born very pre-term. Brain.

[10] Poretti A, Boltshauser E, Doherty D (2014). Cerebellar hypoplasia: differential diagnosis and diagnostic approach. Am J Med Genet C Semin Med Genet.

[11] Panigrahy A, Nelson MD, Bluml S (2010). Magnetic resonance spectroscopy in pediatric neuroradiology: clinical and research applications. Pediatr Radiol.

[12] Ross B, Bluml S (2001). Magnetic resonance spectroscopy of the human brain. The Anatomical record.

[13] Bluml S (2013). Metabolic maturation of the human brain from birth through adolescence: insights from *in vivo* magnetic resonance spectroscopy. Cereb Cortex.

[14] Parikh NA (2016). Advanced neuroimaging and its role in predicting neurodevelopmental outcomes in very preterm infants. Semin Perinatol.

[15] Tomiyasu M (2013). Neonatal brain metabolite concentrations: an *in vivo* magnetic resonance spectroscopy study with a clinical MR system at 3 Tesla. PLoS One.

[16] Moffett JR, Ross B, Arun P, Madhavarao CN, Namboodiri AM (2007). N-Acetylaspartate in the CNS: from neurodiagnostics to neurobiology. Prog Neurobiol.

[17] Demougeot C (2001). N-Acetylaspartate, a marker of both cellular dysfunction and neuronal loss: its relevance to studies of acute brain injury. Journal of neurochemistry.

[18] Van Kooij BJ (2012). Cerebellar volume and proton magnetic resonance spectroscopy at term, and neurodevelopment at 2 years of age in preterm infants. Dev Med Child Neurol.

[19] Jansen JF, Backes WH, Nicolay K, Kooi ME (2006). 1H MR spectroscopy of the brain: absolute quantification of metabolites. Radiology.

[20] Panigrahy A, Borzage M, Bluml S (2010). Basic principles and concepts underlying recent advances in magnetic resonance imaging of the developing brain. Semin Perinatol.

[21] Brossard-Racine M (2017). Cerebellar microstructural organization is impeded by complications of premature birth: A case-control study. Journal of Pediatric.

[22] Kyriakopoulou V (2014). Cortical overgrowth in fetuses with isolated ventriculomegaly. Cereb Cortex.

[23] Benvenisti H, Bassan H, Shiran S, Constantini S, Roth J (2015). “Growing” cerebellum in an infant after shunt insertion. Pediatr Neurol.

[24] Courchesne E (2002). Abnormal early brain development in autism. Mol Psychiatry.

[25] Courchesne E (2004). Brain development in autism: early overgrowth followed by premature arrest of growth. Ment Retard Dev Disabil Res Rev.

[26] Volpe JJ (2009). Brain injury in premature infants: a complex amalgam of destructive and developmental disturbances. Lancet Neurol.

[27] Khwaja O, Volpe JJ (2008). Pathogenesis of cerebral white matter injury of prematurity. Arch Dis Child Fetal Neonatal Ed.

[28] Chau V (2012). Postnatal infection is associated with widespread abnormalities of brain development in premature newborns. Pediatr Res.

[29] Brossard-Racine M (2016). Brain Injury in Neonates with Complex Congenital Heart Disease: What Is the Predictive Value of MRI in the Fetal Period?. AJNR.

[30] Kidokoro H, Neil JJ, Inder TE (2013). New MR imaging assessment tool to define brain abnormalities in very preterm infants at term. AJNR Am J Neuroradiol.

[31] Barkovich, A. Pediatric neuroimagin, Edn. 4th. (Williams & Wilkins, Philadelphie, PA; 2005).

[32] Provencher SW (2001). Automatic quantitation of localized *in vivo* 1H spectra with LCModel. NMR Biomed.

[33] Kreis R, Ernst T, Ross BD (1993). Development of the human brain: *in vivo* quantification of metabolite and water content with proton magnetic resonance spectroscopy. Magn Reson Med.

