# Author Correction: Altered Cerebellar Biochemical Profiles in Infants Born Prematurely

**DOI:** 10.1038/s41598-018-25615-1

**Published:** 2018-05-22

**Authors:** Marie Brossard-Racine, Jonathan Murnick, Marine Bouyssi-Kobar, Janie Coulombe, Taeun Chang, Catherine Limperopoulos

**Affiliations:** 1 McGill University Health Centre, Division of Pediatric Neurology, Montreal, PQ Canada; 2 School of Physical and Occupational Therapy, McGill University, Montreal, PQ Canada; 3 Division of Diagnostic Imaging and Radiology, Children’s National Health System, Washington, D.C. USA; 4 Developing Brain Research Laboratory, Children’s National Health System, Washington, D.C. USA; 5 Epidemiology, Biostatistics and Occupational Health, McGill University, Montreal, PQ Canada; 6 Neurophysiology, Epilepsy and Critical Care, Children’s National Health System, Washington, D.C. USA

Correction to: *Scientific Reports* 10.1038/s41598-017-08195-4, published online 15 August 2017

The Acknowledgements section in this Article is incomplete.

“We would like to thank the families who participated in the study as well as all the MRI & NICU nurses, MRI technologists, and physicians at Children’s National Health System for their assistance with the study. We also would like to thank Manouchka Jean-Gilles, PhD, for her assistance with manuscript editing. This work was supported by the Canadian Institutes of Health Research (MOP-81116). Dr. Brossard-Racine received fellowship support from the Canadian Institutes of Health Research at the time of data collection and analyses.”

should read:

“We would like to thank the families who participated in the study as well as all the MRI & NICU nurses, MRI technologists, and physicians at Children’s National Health System for their assistance with the study. We also would like to thank Manouchka Jean-Gilles, PhD, for her assistance with manuscript editing. This work was supported in part by the Canadian Institutes of Health Research (MOP-81116) and by the Intellectual and Developmental Disabilities Research Centers Award (NICHD-P30HD40677) from the Eunice Kennedy Shriver National Institute of Child Health and Human Development. Dr. Brossard-Racine received fellowship support from the Canadian Institutes of Health Research at the time of data collection and analyses.”

